# The Role of Adipose-Derived Stem Cells, Dermal Regenerative Templates, and Platelet-Rich Plasma in Tissue Engineering-Based Treatments of Chronic Skin Wounds

**DOI:** 10.1155/2020/7056261

**Published:** 2020-01-09

**Authors:** Massimo Conese, Luigi Annacontini, Annalucia Carbone, Elisa Beccia, Liberato Roberto Cecchino, Domenico Parisi, Sante Di Gioia, Fedele Lembo, Antonella Angiolillo, Filiberto Mastrangelo, Lorenzo Lo Muzio, Aurelio Portincasa

**Affiliations:** ^1^Department of Medical and Surgical Sciences, University of Foggia, Foggia 71122, Italy; ^2^Department of Clinic and Experimental Medicine, University of Foggia, Foggia 71122, Italy; ^3^Unit of Reconstructive and Plastic Surgery, Ospedali Riuniti di Foggia, Foggia 71122, Italy; ^4^Division of Internal Medicine and Chronobiology Unit, IRCCS “Casa Sollievo della Sofferenza”, San Giovanni Rotondo, Foggia 71013, Italy; ^5^Department of Medicine and Health Sciences “V. Tiberio”, University of Molise, Campobasso 86100, Italy

## Abstract

The continuous improvements in the field of both regenerative medicine and tissue engineering have allowed the design of new and more efficacious strategies for the treatment of chronic or hard-to-heal skin wounds, which represent heavy burden, from a medical and economic point of view. These novel approaches are based on the usage of three key methodologies: stem cells, growth factors, and biomimetic scaffolds. These days, the adipose tissue can be considered the main source of multipotent mesenchymal stem cells, especially adipose-derived stem cells (ASCs). ASCs are easily accessible from various fat depots and show an intrinsic plasticity in giving rise to cell types involved in wound healing and angiogenesis. ASCs can be found in fat grafts, historically used in the treatment of chronic wounds, and have been evaluated as such in both animal models and human trials, to exploit their capability of accelerating wound closure and inducing a correct remodeling of the newly formed fibrovascular tissue. Since survival and fitness of ASCs need to be improved, they are now employed in conjunction with advanced wound dressings, together with dermal regenerative templates and platelet-rich plasma (as a source of growth and healing factors). In this work, we provide an overview of the current knowledge on the topic, based on existing studies and on our own experience.

## 1. Introduction

Tissue engineering is an interdisciplinary field of biomedical research which focuses on the restoration of tissue defects, up to the replacement of a complete organ [1, 2]. A well-established approach for the generation of tissue is based on cells seeding onto different biomaterials, serving as three-dimensional scaffolds. To promote the function and the regenerative capability of the cells which have been seeded, scaffolds should mimic the natural extracellular matrix [3–6]. To reach this goal, three components are needed: (1) stem cells, (2) a scaffold supporting the formation of both tissue architecture and cell function, and (3) an appropriate cocktail of growth factors and other molecules with trophic, surviving, and proangiogenic properties [7].

Adult stem cells are multipotent and therefore able to differentiate into a limited number of cell types, often those originating from the same germ layer. One type of adult somatic stem cells is represented by mesenchymal stem cells (MSCs), deriving from the mesodermal embryonic tissue, showing self-renewal capability and multipotent ability (i.e., they are precursors of cartilaginous, osseous, adipose, and other mesenchymal tissues [8]). Even if the bone marrow is the most common source for MSCs, they have been identified as well in skeletal muscles, pancreas, synovium, skin, blood vessels, adipose tissue, and placenta [9, 10]. It has been shown that MSCs isolated from different sources share similar characteristics. Recently, it has been recognized that subsets of MSCs with differences in protein and gene expression can be identified in the various tissues [11].

One of the main sources of stem cells is adipose tissue, which, in recent studies, has been reported to contain multipotent and pluripotent stem cells able to regenerate themselves and to differentiate in a variety of specialized cell types [12, 13]. Due to its abundance and accessibility by means of minimal-invasive techniques, adipose tissue represents an attractive source for harvesting stem cells, with minimum discomfort for the patient. Autologous fat transfer (also called “lipofilling”), in fact, has proven to be an easy technique, minimally invasive, and which can be completed in an outpatient way. It has been used for in a variety of indications, including treatment of tissue discontinuities, burns, scars, restoration of either facial or body volume in cosmetic surgery, breast reconstruction or aesthetic surgery, and treatment of difficult wounds [14–16].

The low survival rate of adipocytes and ASCs in the wound site has triggered the creation of adjunct dressings. These dressings are not only meant to increase the ASC survival but also meant to promote their proliferation, differentiation, and paracrine abilities [17].

In this study, we aim to give an overview about the role of ASCs, dermal regeneration templates, and growth factors as supplied by platelet-rich plasma (PRP) in the treatment of skin wounds having different etiology.

## 2. Adipose-Derived Stem Cells

ASCs have been shown to have the same biological capabilities as Bone Marrow Mesenchymal Stem Cells (BM-MSCs) [9]. The advantages of ASCs over BM-MSCs and other adult stem cell types are that ASCs are relatively easy to obtain from liposuctions performed in local anesthesia, they can be obtained in large numbers, they are capable of maintaining their phenotype and plasticity after a long-term in vitro culture, and they show low immunogenicity [18]. In particular, ASCs express a low level of major histocompatibility complex (MHC) class I molecules and do not express MHC class II and costimulatory molecules, such as CD40, CD80 (B7-1), and CD86 (B7-2) [19–21]. Interestingly, also adipogenic-differentiated ASCs have no immunogenicity and are clinically safe [22]. Based on these properties, ASCs have generated great interest and are perceived as the most preferred cell type for tissue engineering and regenerative medicine [23].

Zuk et al. [24] were the first to investigate whether human adipose could be an alternative source of MSCs. The authors obtained human adipose tissue from liposuction aspirate and used collagenase to release stromal cells from the extracellular matrix by processing the so-called stromal vascular fraction (SVF), containing a variety of different types of cells including ASCs. The isolated adipose stromal cells were cultured with defined media to induce adipogenic, osteogenic, or chondrogenic differentiation. It was observed that adipose stromal cells could develop intracellular lipid stores, alkaline phosphatase expression, or proteoglycan expression—markers indicative of adipose, bone, and cartilage tissues, respectively. In order to determine if the isolated adipose stromal cells were indeed stem cells, Zuk et al. [13] examined the surface antigen expression and differentiation capacity of clonogenic cultures. Using flow cytometry, the authors observed that the clonogenic cells expressed surface antigens, similar to marrow MSCs. Since BM-MSCs have been the gold standard for MSCs for many years, most of the properties of ASCs have been described through a comparison with marrow MSCs. Following the findings of Dominici et al. [25], BM-MSCs and ASCs are shown to share a fibroblast-like morphology and immunophenotype for mesenchymal markers [26]. Like BM-MSCs, ASCs express higher levels of stromal-associated markers (CD13, CD29, CD44, CD73, CD90, CD105, and CD166) by the later stages of culture [27]. A position paper issued jointly by the International Federation for Adipose Therapeutics and Science (IFATS) and the International Society for Cellular Therapy (ISCT) added that ASCs can be distinguished from BM-MSCs by their positivity for CD36 and negativity for CD106 [28]. Moreover, freshly isolated ASCs present the expression of the stem cell-associated marker CD34 at higher levels than BM-MSCs, which gets eventually lost upon culture [29]. Nevertheless, ASCs and BM-MSCs differ concerning the isolation yield, the frequency of colony-forming units, and the differentiative capacity. ASCs are usually isolated from the SVF of the adipose tissue [13, 24] in numbers which range from 40 to 100-500-fold higher than BM-MSCs [30, 31]. Moreover, it appears that ASCs and BM-MSCs display some differences at genomic and proteomic levels, as well at functional level [32–35]. Finally, meanwhile BM-MSCs are more committed towards the osteoblastic and chondrogenic lineages, ASCs are prone to stimulate angiogenesis [36, 37]. ASCs present also an increased proliferative rate and osteogenic differentiation capacity as compared with dental pulp stem cells (DPSCs) [38].  Table 1 presents the main phenotypic characteristics of ASCs.

### 2.1. ASCs and Fat Grafting

Lipofilling is a low-risk procedure that can be used to correct soft-tissue defects in the face, trunk, and extremities, with minimal discomfort for patients. The clinical applications of autologous fat graft are many, including the treatment of burn scars and radiodermatitis [41]. However, there is no common agreement concerning the ideal harvesting (suction vs. resection) and fat processing methods [42–44]. In 1987, Coleman introduced a new technique to decrease traumatic handling of fat during liposuction. Coleman's technique consisted of three steps: manual lipoaspiration under low pressure, centrifugation at low speed, and reinjection in small aliquots in a “fanning-out” pattern to varying depths in the soft tissue, only during withdrawal of the cannula [45–48]. One of the problems observed in the process is related to the decrease in the number of fat cells, caused by the damage during the aspiration and centrifugation steps [49], as well as ischemic conditions at the injection site [50]. In the long term, an unpredictable absorption rate of up to 70% of the volume of the fat graft has been reported [43]. Another limitation of fat grafting is the requirement to infiltrate cells in direct contact with well-vascularized tissues [49]. Despite the technical limitation, it is important to highlight how not only fat is a filler with 100% biocompatibility but it also contains the SVF, which is composed by a heterogeneous population of many cell types, including preadipocytes, endothelial cells, pericytes, haematopoietic-lineage cells, and fibroblasts. ASC frequency in the SVF is nearly 2%, considered to be the highest among all kinds of tissues [51]. Because of their properties and because these cells can be easily harvested in great amounts with minimal donor-site morbidity, ASCs have proved to be particularly promising for enhancing the efficacy of fat grafting. Cell-Assisted Lipotransfer (CAL) is the coinjection of fat and ASCs that has proven to prolong fat survival at the site of injection and provide cues for neoangiogenesis in vivo [52]. Neoangiogenesis is considered the main condition key to ensure fat graft survival. As a consequence, CAL has been successfully used in several clinical indications, including hemifacial atrophy (Parry-Romberg disease) [53], breast augmentation [54], and replacement of breast implants [55].

Factors such as donor age, adipose tissue type (white or brown), and anatomical location (subcutaneous or visceral adipose tissue) might influence the functional behaviour of ASCs. The abdomen is the most common site of fat harvesting, followed by the trochanteric region (saddlebags) and the inside of the thighs and knees [41]. Schipper and colleagues [56] have demonstrated that sensitivity to apoptosis was linked to anatomic depot, with the superficial abdominal depot being statistically significantly less susceptible to an apoptotic stimulus when compared with the deep abdominal, arm, thigh, and trochanteric depots. On the other hand, young patients (25-30 years old) showed a higher proliferation rate than older donors. As to the differentiation capacities of ASCs, two studies have highlighted that abdominal subcutaneous ASCs display less osteogenic potential than other subcutaneous depots [57, 58]. In general, subcutaneous ASCs from the thigh/gluteus/flank appeared to be most primed to undergo osteogenesis, while, compared to other adipose depots, including visceral, omental, and intrathoracic, subcutaneous ASCs have consistently displayed greater osteogenic potential [59]. This finding has been confirmed in a recent study, which shows a better performance of ASCs coming from subcutaneous adipose tissue vs. those from visceral fat tissue in giving rise to bone aggregates in vitro [60]. The role of gender and fat depot (superficial and deep adipose layers of the abdominoplasty specimens) was investigated by Aksu et al. [61], who found that there was no significant difference in the degree of osteogenic differentiation between the ASCs from either depots in females. In the male group, the superficial depot ASCs differentiated faster and more efficiently than those of the deep depot. Male ASCs from both depots differentiated more effectively than female ASCs from both depots. An extensive comparison study on abdominal subcutaneous fat and omentum, pericardial adipose tissue, and thymic remnant samples has revealed that ASCs isolated from both intrathoracic depots had a longer average doubling time than ASCs from the subcutaneous/omentum donors [62]. Moreover, subcutaneous ASCs demonstrate enhanced adipogenic differentiation relative to ASCs derived from the omentum, whereas osteogenic differentiation was enhanced in omentum ASCs. Noteworthy, in vitro clonogenic potential and osteogenic differentiation were enhanced under hypoxic condition for all depots. All-in-all, these observations point out to the necessity of considering the choice of fat depot as an important factor when evaluating clinical application in tissue engineering.

### 2.2. Angiogenesis and ASCs

ASCs are also prone to stimulate angiogenesis [36, 37], an essential step for regenerative purposes. They can give rise to vascular cells, both smooth-muscle cells and endothelial cells [63–65]. They have also been shown to be able to differentiate into cells of ectodermic leaflet such as neuronal cells [39, 66–68]. Their neurotrophic and angiogenic properties were reported to be due to the secretion of nerve growth factor (NGF), brain-derived neurotrophic factor (BDNF), glial cell-derived neurotrophic factor (GNDF), vascular endothelial growth factor-A (VEGF-A), and angiopoietin-1 [69]. Indeed, the secretome of ASCs is complex; ASCs have the property to secrete proteins involved in angiogenesis, wound healing, tissue regeneration, and immunomodulation [70]. Moreover, ASCs have been shown to secrete anti-inflammatory and immunomodulatory mediators, such as prostaglandin E2 and indoleamine 2,3-dioxygenase [21, 71]. Interestingly, a recent work on ASCs in pediatric patients has shown their ability to give rise to nonmesenchymal cells with significant plasticity. Indeed, mesenchymal lineages (adipogenic, chondrogenic, and osteogenic) and also neural and epithelial lineages can originate from clonal lines that like the parental line express markers of pluripotency [72]. Thus, ASCs are endowed with properties considered essential in wound healing and can be considered to contribute to the improvement of hard-to-heal wounds, not effectively treated by current drugs or surgery [17].

In line with the data reported, ASCs were shown to be associated with the presence of small vessels. Based on the positivity to the CD34 antigen, Zimmerlin and colleagues [73] identified two different perivascular subpopulations in SVF: pericytes (CD146^+^/CD34^−^/CD31^−^) and the so-called supraadventitial adipose stromal cells, SA-ASC (CD146^−^/CD34^+^/CD31^−^). Other studies have shown that pericytes and ASCs share many cell surface markers, including smooth muscle *β*-actin, platelet-derived growth factor (PDGF) receptor-*β*, and neuroglial proteoglycan 2 [74, 75], thus bringing to hypothesize that pericytes could be precursor cells for ASCs *via* a transitional cell [73]. Previously, Lin and colleagues [76] had found that ASCs exist as CD34^+^/CD31^−^/CD104b^−^/*α*-SMA (alpha-smooth muscle actin)^−^ cells in the capillaries and in tunica adventitia of larger vessels in vivo, suggesting that in the capillary, these cells coexist with pericytes and endothelial cells, both of which are possibly progenies of ASCs, while in the larger vessels, these ASCs exist as specialized fibroblasts (having stem cell properties) in the adventitia.

### 2.3. Isolation and Differentiation of ASCs

Many challenges in the use of SVF and ASCs need to be overcome before these products can be fully exploited in tissue engineering and in an extensive usage in regenerative medicine. One of the challenges concerns the isolation method, which can have effects on the phenotype and functional properties of ASCs. The most widely used technique for ASC isolation is represented by digestion of the lipoaspirate (usually with collagenase) to isolate the SVF from the buoyant adipocytes [24, 52]. However, the presence of collagenase in the injectable product seems to be difficult to be approved by authorities such as the FDA. Therefore, alternative methods, i.e., mechanical forces to isolate SVF are being explored [77–79]. When compared with enzymatic methods, the cellular yield from mechanical procedures is much lower [78, 80], even though an enrichment in progenitor phenotypes consistent with multipotency and pluripotency is noted [81].

Tonnard et al. [82] compared three methods of fat harvesting: macrofat, microfat, and nanofat. Macrofat and microfat were achieved with a standard cannula with large side holes (3 × 7 mm) and a multiperforated cannula with sharp side holes of 1 mm in diameter, respectively. Nanofat was obtained further processing the microfat by mechanical emulsion and filtration. Interestingly, both macrofat and microfat showed good adipocyte viability and adipose tissue structure in contrast with the nanofat, where no adipocytes and no normal adipose tissue structure were visible. Nevertheless, all three types of samples contained 5.1-6.5% of CD34^+^ cells on the total of all SVF cells, implying that the emulsification/filtration step did not hamper the presence of good numbers of ASCs in the nanofat grafting. The efficacy and the key properties of nanofat have been demonstrated in multiple case studies, concerning skin rejuvenation [83], scar healing [82], skin grafting for wound management [84], and the treatment of vulvar lichen sclerosus (VLS), a chronic inflammatory disease of the anogenital area [85]. The combination of the emulsification technique with the centrifugation phase can yield products highly concentrated in ASCs, eliminating therefore enzymatic digestion, reducing process time and cost, and acting on regulatory constraints [86]. Our group produced interesting results by combining emulsification with enzymatic digestion: we obtained abdominal subcutaneous adipose tissue via an aesthetic liposuction procedure and we subjected half of it to enzymatic digestion with type I collagenase. We subjected the other half to emulsification and then to collagenase digestion. Emulsification was performed accordingly with Tonnard et al. [82], thus by shifting the fat between two 10 cc syringes connected to each other by a female-to-female Luer-Lok connector for 30 times. By using this procedure, the yield of SVF cells reached was almost threefold higher, as compared with enzymatic digestion only (unpublished results). Others have shown that mechanical combined with enzymatic digestion allowed to isolate a greater number of ASCs (25.9% of the total number of harvested cells) compared to the mechanical protocol (5%) [87]. It is also noteworthy to quote a recent publication in which the authors have demonstrated that mechanical isolation by intersyringe shuffling gave a higher yield of ASCs in comparison with collagenase digestion [88]. Although mechanical emulsification is a more suitable (and cheaper) procedure, in the light of improved yield of ASCs with combination of mechanical protocol and collagenase digestion, it would be interesting to validate this double application. However, the appropriate concentration, inactivation, and method for washing collagenase have not been determined yet. One study demonstrated that residual collagenase activity is minimal after appropriate ASC washing and that no specific toxicity as a result of residual collagenase was observed in a 4-week toxicity study in BALB/c-nu mice [89]. In alternative, a GMP-grade collagenase is ought to be used [90].

The differentiation of ASCs towards mesenchymal and nonmesenchymal lineages has also been questioned. For example, De Ugarte et al. [91], by comparing ASCs and BM-MSCs, found no difference in the number of cells developing lipid droplets (adipogenic cells), or the alkaline phosphate activity of osteogenic cells. However, when induced to differentiate into cartilage, adipose-derived cells stained positive for chondrogenesis while marrow-derived cells did not. Using similar methods, other investigations have compared the ability of marrow and adipose MSCs to differentiate along these lineages and have demonstrated that cells from either tissue possess an equal capacity to become adipose, bone, and cartilage [34]. Overall, these results may have been affected by different culture conditions and/or the isolation of different subsets of MSCs, highlighting that the potential of ASCs to differentiate into either osteoblasts or chondrocytes remains controversial. Although, as cited above, ASCs and BM-MSCs show some differences in the differentiation capability, ASCs display the same capacity of BM-MSCs to give rise in vivo to osteophytes and chondrophytes when injected in the knee of severe combined immunodeficient (SCID) mice [35]. Thus, it seems likely that environmental factors present *in vivo* are important for proper differentiation towards mesenchymal lineages of ASCs. This is also the take-home message of our recent work [92] that is described by cytochemistry of the differentiative capacities of ASCs. When ASCs were directed to differentiate into adipocytes, they readily did so. However, after 2-4 weeks of induction, ASCs failed to differentiate into either osteoblasts or chondrocytes—they just changed morphology without producing either calcium or proteoglycans. The picture changed when ASCs were treated with conditioned medium obtained from cultures of either DPSCs induced to osteoblasts or prechondrocytes induced to differentiate into chondrocytes—they acquired the phenotype of osteoblasts and chondrocytes. These results point out to the relevance of paracrine factors secreted by differentiated cells of the tissue microenvironment.

More recently, stimulated ASCs seeded onto fibrin conduits were shown to boost axon regeneration and angiogenesis in a rat sciatic nerve injury model [69], although the precise mechanism of neurite outgrowth mediated by ADSC remains to be elucidated.

Recent studies have shown that human ASCs used in animal experimental models can promote tissue formation and enhance graft retention as a result of enhanced vascularity [93–95]. In this context, however, the precise mechanisms are not known. It is possible to assume a synergy between engraftment of ASCs into the novel vessels and paracrine factors. Choi and colleagues [94] showed that coimplantation of ASCs with rat cardiomyocytes in a vascularized tissue engineering chamber resulted in integration of ASCs into the endogenous vascularization of the construct. On the other hand, the same group demonstrated that while increased levels of the proangiogenetic chemokine interleukin- (IL) 8 were observed *in vitro* when ASCs were grown on a rat cardiac extracellular matrix (ECM) extract-derived hydrogel (cardiogel), the presence or absence of cardiogel had no effect on neoangiogenesis in the tissue chamber model [93]. In general, these results suggest that the right combination of ASCs and ECM scaffold should be further explored for tissue engineering purposes *in vivo*.

Taken together, the above results demonstrate that, compared to bone marrow, a high number of MSCs capable of multilineage differentiation can be obtained from adipose tissue and that more cues to their differentiation should be found recapitulating *in vivo* situation.

Due to their interesting properties, ASCs have been found useful for plastic surgery applications, including fat grafts, management of hard-to-heal wounds, regeneration of local soft tissue defects, bone reconstruction, recovery from acute tissue ischemia of vascular origin, and scar management [96–98]. The optimal delivery system has to be tailored for each indication, which caused a variety of techniques to be tested for ASC treatment, including systemic administration, local injection, topical applications, and different scaffolds [98].

### 2.4. Application of ASCs for Chronic Wound Healing in Preclinical Models

Chronic wound healing represents one of the major challenges in plastic surgery, and as yet, no satisfactory therapy has been identified. Some positive results have been obtained in preclinical animal models [99] showing how chronic wounds could benefit greatly from the application of ASCs, even if many challenges remain to be handled, including ASCs delivery techniques, dosages, and isolation procedures.

#### 2.4.1. Diabetic Wounds

Some interesting studies have been performed in diabetic animal models. Amos et al. [100] evaluated the administration of ASCs to full-thickness dermal wounds in *db/db* mice by comparing self-organised multicellular aggregates (MAs) to cell suspensions. Application of MAs decreased the wound size faster than ASCs in suspension, while the healing rates of mice treated with MAs were similar to those of nondiabetic control mice. Interestingly, this 3D culture of ASCs increased the mRNA levels and secretion of several factors involved in angiogenesis and ECM delivery, production, and remodeling (like insulin-like growth factor binding protein-1, transforming growth factor-*β*1 (TGF-*β*1), VEGF, hepatocyte growth factor (HGF), decorin, biglycan, and fibronectin), highlighting the role of ECM in the *in vivo* therapeutic properties of ASCs.

Maharlooei et al. [101] showed that ASCs were able to accelerate wound-healing rate in diabetic rats, but did not increase length and volume density of the vessels and volume density of the collagen fibers. Furthermore, ASCs decreased the numerical density of fibroblasts. These data suggested that ASC-dependent healing effect does not depend on transdifferentiation, rather on other mechanisms which may be underlining. Another work [102] showed that ASCs significantly promoted wound-healing process in a rat model of excisional wound healing and that they reduced the time required for complete closure in both normal and diabetic rats. Furthermore, Green Fluorescent Protein- (GFP) positive ASCs appeared to express *in vivo* epithelial and endothelial markers, suggesting that ASCs can accelerate wound healing by both contributing to dermal keratinocytes and neovascularization process. Moreover, a strong VEGF and HGF protein expressions were observed in ASC-treated wounds, while they were lowly expressed in the control and fibroblast-treated wounds. In line with these last results, in an *in vitro* wound-healing assay, Lee et al. [103] demonstrated that treatment with MSCs significantly increased the proliferation of HaCaT (immortalized human keratinocyte) cells and dermal fibroblasts and increased overall wound healing.

Kim and colleagues [104] determined that in a diabetic mouse model, wounds treated with amnion-derived (AM) MSCs displayed accelerated wound healing at days 7, 10, and 14, compared with those treated with ASCs or human dermal fibroblasts. These wounds also displayed enhanced reepithelialization compared with wounds treated with ASCs. These results are likely explained by the increase engraftment of amnio-derived MSCs as compared to ASCs. Of note, contrasting results were seen by Liu et al. [105], who reported that human ASCs had the most pronounced effect on wound closure, followed by the human BM-MSCs, human AM-MSCs, and the control groups, and were associated with the greatest reepithelialization and the thickest granulation tissue. ASCs also had the greatest effect on human dermal fibroblast migration and expression of type I collagen. Moreover, fibroblasts cultured with ASCs had significantly higher expression of VEGF, basic fibroblast growth factor (bFGF), and TGF-*β*. Therefore, ASCs are for sure endowed with interesting properties for inducing would healing; however, future investigations into which source provides MSCs that exhibit the greater improvement in wound healing and therapeutic efficacy are needed.

Autologous transplantation of ASCs in a full-thickness wound model in diabetic rats was studied by Zografou and colleagues [106]. ASC injection into the wound bed determined increased survival, angiogenesis, and epithelialization as compared with untreated controls. Also, increased capillary and collagen density as well as increased expression of VEGF and TGF-*β*3 (as antiscarring factor) in ASC-treated rats was observed.

Kim and colleagues [107] performed a study comparing normal ASCs to diabetic ASCs in a wound-healing model created by including a silicone layer in the artificial dermis in order to minimize skin contraction around the wound site. At 1 week, the wound-healing rate was significantly higher in the normal ASC group than in the diabetic ASC group and the untreated control group. Similar results were observed for reepithelization and keratinocyte proliferation, granulation tissue formation, and dermal regeneration. Despite diabetic ASCs retained their ability to promote neovascularization and angiogenesis in this setting, one may assume that it may be better to employ allogenic normal ASCs rather than autologous diabetic impaired ASCs in cell therapy for promotion of wound healing in diabetic patients. The low immunogenicity of ASCs, combined with their anti-inflammatory potential, thus limiting the host immunoreaction against themselves in case of allogeneic transplantation, would be proficient for the allogenic use of ASCs in the context of diabetic ulcers. This evidence is still to be gained in ongoing clinical trials and should be validated in future clinical studies [108, 109].

#### 2.4.2. Burn Wounds

Another clinical challenge for plastic surgery is represented by severe burns. Current treatment options for severe burn wounds are often insufficient in reconstructing skin and soft tissue defects. Two groups investigated the potential application of ASCs for the treatment of burn wounds. Loder et al. [110] demonstrated that three types of grafts, processed adipose tissue, ASCs only, or mixed adipose isografts, would improve certain aspects of wound healing in a mouse model of burn injury. In particular, they showed that fat isografts remain viable through the early period of wound healing when placed at the burn site, that burn wound depth and area were reduced in mice which have been treated with fat and/or ASCs, and, finally, that apoptotic markers were significantly reduced in mice receiving treatments that included ASCs. In Bliley et al.'s work [111], injection of concentrated ASCs (6.8 × 10^6^ cells) into a burn wound enhanced the vascularity and type I and III collagen deposition. Moreover, the administration of ASCs enhanced the adipocyte differentiation within the burn wound, indicating that these cells have the potential to initiate the adipogenic pathway in native tissues.

#### 2.4.3. Radiation Ulcers

Radiation ulcers can be a nasty side effect of cancer patients receiving radiation as an adjuvant therapy. Huang et al. [112] showed that local injection of ASCs promoted a faster wound healing as compared with control in radiation ulcers induced in the dorsum of rats with an electronic beam, and the granulation tissue and blood supply could as well be increased by ASC injection.

#### 2.4.4. Surgical Wounds

Wound healing in pediatric patients is of great clinical and social importance, since scarring can have severe aesthetic and psychological consequences from infancy to late adolescence. The data obtained from preclinical study using young rabbits performed by Pelizzo and her group [113] could be translated to regenerative medicine for the treatment of congenital and acquired skin lesions occurring in the pediatric age. At certain times during childhood, such as in neonates or young infants, adipose tissue harvesting may be difficult or limited. Therefore, further studies focusing on the potential efficacy of cell therapy approaches in this particular area with the use of allogeneic ASCs obtained also from adult donors or cell-free extracellular vesicles [114] are urgently needed.

Table 2 summarizes the preclinical studies about the usage and results obtained with ASCs in the field of wound healing [101–107, 110–113, 115–117].

These preclinical studies, supporting the therapeutic role of ASCs in the treatment of chronic wounds, have been followed by clinical studies applied to different fields, including plastic surgery [118]. Autologous ASCs were reported to be effective in the regeneration of widespread traumatic calvarial bone defects [119], cranio-maxillofacial hard-tissue defects [120], breast augmentation or reconstruction [121, 122], and chronic ulcers caused by radiation therapy [122], as well as in chronic wounds [121].

## 3. Dermal Regeneration Templates

Skin is the largest organ of the body with many essential functions. Due to its direct contact with the external environment, which makes it extremely prone to damage and/or injury, the skin plays a crucial role as a barrier against exogenous substances, pathogens, and mechanical stresses. Damages to this barrier lead to loss of water and proteins, and bacterial invasion to the underlying tissue. Hence, a quick regeneration after injury is deemed to avoid complications [123], and a wide range of biomaterials has been used by the medical practitioners to manage the chronic wounds [124, 125].

Many efforts have been made by the researchers to promote the regeneration of the skin, and many studies demonstrate the usefulness of either allografts or autografts. For this purpose, different polymeric biomaterials were developed, which can act as smart “skin substitutes” by performing many of skin's functions and are made with varied combinations of synthetic and/or biologic substances. Depending on the product characteristics, skin substitutes could replace skin either temporarily or permanently [126]. These devices are an alternative to the standard wound coverage in circumstances when standard therapies are not applicable [127]. Skin substitutes are used to help wound healing, alleviate pain and replace the function of the skin, and have an important role in the treatment of deep dermal and full thickness injuries of various etiologies [128].

It is possible to recognize three types of skin substitutes from which skin layer they are formed of: those consisting only epidermal equivalents, those involving dermal components from processed skin, and those made both of dermal and epidermal components [129]. Skin substitutes can be made of human tissue (allografts), animal tissue (xenografts), or using membranes developed from natural or synthetic polymers. Even if there is no ideal skin substitute available, tissue engineering and bioengineering are trying to create an ideal one with all the best properties shown by each available device. Biological skin substitutes have the advantage of being relatively abundant in supply and present a more intact and native ECM structure, showing excellent reepithelialization characteristics [128]. The most widely used biological substitutes worldwide are cadaveric skin allograft, porcine skin xenograft, amnion, and cultured epithelial autografts (CEA), with their own characteristics and limits [128]; synthetic skin substitutes are constructed from nonbiological molecules and polymers that are not present in human skin [130]. These constructs should be safe, stable, and biodegradable and provide an adequate environment for the regeneration of tissue. Moreover, biodegradation should preferably take place after this period.

Acellular skin substitutes may consist of either biopolymers, such as collagen and chondroitin-sulfate or elastin (Integra® Dermal Regeneration Template (IDRT) [131]; Matriderm® [132]), decellularized human dermis (AlloDerm®) [133], derivatized hyaluronic acid (Hyalomatrix®) [134], or polyurethane (BioTemporizing Matrix, “BTM”) [135, 136]. Each of these materials protects open wounds, promotes ingrowth of fibrovascular tissue, and may suppress granulation tissue and scar. In order to foster and accelerate wound healing by these skin substitutes, various differentiated cell types have been added to these substitutes, including keratinocytes [137, 138], melanocytes [139, 140], microvascular endothelial cells [141–143], sensory nerve cells [144], and differentiated adipocytes [145]. Also, stem/progenitor cells such as hair follicle progenitor cells [146, 147], induced pluripotent stem cells [148, 149], and mesenchymal stem cells [150], including ASCs [151–163], have been used (Table 3). The application of an acellular dermal matrix (ADM) with ASCs in skin injury models [151, 152, 155, 160] determined that ASCs survived in vivo, spontaneously differentiated along vascular, fibroblastic, and epidermal lineages, and increased vascularity of the implants as well as improved wound healing. Following studies showed that ADM containing ASCs, previously grown in vitro in autologous plasma-supplemented medium, promoted the neovascularization and reepithelization within the time frame of 12 days [95]. Increased vascularization and collagen deposition was observed also when ASCs were seeded into Integra® either in a rat model of skin wound [156] or when the scaffolds were implanted subcutaneously in the dorsum of nude mice [159].

Our group has a long-term experience concerning integrated approaches using myocutaneous flaps, lipofilling, and Integra®, a bilayer membrane system for skin replacement [16, 164, 165]. This dermal replacement layer is made of a porous matrix of fiber of cross-linked bovine tendon collagen and glycosaminoglycan (chondroitin-6-sulfate) manufactured with a controlled porosity and defined degradation rate [166]. The epidermal substitute layer is made of thin polysiloxane (silicone) layer to control moisture loss from the wound. We have treated a limited number of patients (*n* = 9) with Integra® and an autologous thin dermal-epidermal graft for losses of skin tissue and subsequently with lipofilling [165]. We detected an overall clinical and histological improvement in all cases. Immunohistochemistry with CD31 antibody also demonstrated quantitative changes with an increased number of vessels. Masson trichomic staining also showed newly formed immature collagen after lipofilling.

Integra® is frequently used as scaffold material for experimental tissue engineering studies, because it easily enables cellular seeding due to its porous structure with pore sizes ranging between 20 and 125 *μ*m [167]. Moreover, it shows specific resistance to degradation by collagenase, and its in vivo degradation rate is 30 days. Currently, as shown also from significative clinical evidences, Integra® IDRT can be considered the only ADM able to induce neodermis formation while other scaffolds composed of derivatized hyaluronic acid, or only collagen of porcin or bovine origin, scaffolds of synthetic origin and scaffolds of biosynthetic origin, must be considered “bioinductors” of granulation tissue since they act only temporary for a too short time and are degraded too rapidly. These differences between Integra® IDRT to have a controlled half-life of 14 ± 7 days and therefore the ability to create neodermis, compared to the other only collagen composed scaffolds, are related to the presence of the component chondroitin-6-sulfate. It causes the structural integrity of the tissue and allows interactions between cells and ECM, increases the resistance of the scaffold against the action of collagenases, has anti-inflammatory properties, and presents a specific surface chemistry that leads to an optimal interaction between the scaffold and the cells (e.g., myofibroblasts) responsible of creating new dermis [168]. In our experience, Integra® is a suitable scaffold for colonization and survival by ASCs. A dose- and time-dependent study showed that passage 2-ASCs survived until 15 days when seeded at least in the number of 1 × 10^6^ considering a 1 × 1 cm piece of Integra® (unpublished results).

## 4. Platelet-Rich Plasma

The current practice of regenerative medicine applied to the treatment of chronic wounds encompasses not only the use of mesenchymal stem cell therapy but also platelet-rich plasma (PRP), a concentration of blood-derived human platelets in a small volume of plasma. PRP has been widely used across many clinical fields, especially for skincare and aesthetic surgery. The key feature of this technique is linked to the fact that platelets are deeply involved in wound healing. During the formation of the primary clot, platelets release growth factors and cytokines that stimulate cell proliferation, differentiation, and neoangiogenesis [70, 169, 170]. PRP contains many growth factors, including epidermal growth factor (EGF), platelet-derived growth factor (PDGF), TGF-*β*, VEGF, FGF, insulin-like growth factor (IGF), and keratinocyte growth factor (KGF), all having important roles in wound healing and tissue regeneration [171, 172]. Moreover, PRP stimulates the expression of type I collagen and matrix metalloproteinase-1 (MMP-1) in dermal fibroblasts [173] and increases the expression of G1 cycle regulators, type I collagen, and MMP-1 to accelerate wound healing [174]. Several reports have adjunctively demonstrated that PRP increases ASC proliferation. Cervelli et al. showed that PRP induced an increase in ASC number by threefold-fourfold without any morphological changes [169]. No differences were noted in adipogenic differentiation between PRP-treated and control ASCs [169]. This effect was dose-dependent with an EC50 of 15.3 ± 1.3% vol/vol [175]. Interestingly, Van Pham and colleagues obtained similar results in the proliferation assay, determining that 15% PRP was the optimal concentration for robust proliferation of ASCs [176]. More recently, it has been shown that ASCs grown in 10% PRP proliferated faster and were healthier and smaller with normal fibroblast-like morphology as compared with those cultured in 10% FBS [177].

PRP has been used in combination with fat grafting for the treatment of lower extremity ulcers, with 61.1% and 88.9% undergoing to 100% reepithelization after a 7.1-week and 9.7-week twice-daily course, respectively, compared with 40% and 60% of the control group, treated with hyaluronic acid and collagen medication [169]. In a subsequent study, it was shown that patients affected by posttraumatic lower extremity ulcers and treated with PRP associated with fat grafting displayed an improvement in reepithelialization; in fact after 9.7 weeks, they underwent a 97.8 ± 1.5% reepithelialization compared to 89.1 ± 3.8% of the control group (only PRP) [178]. The efficacy of PRP mixed with fat grafting in the treatment of chronic-lower extremity ulcers [179] and loss of substance on the lower limbs [180] has been further demonstrated.

In order to recruit endogenous dermal stem/progenitor cells from the tissue surrounding the injury, PRP has been used in combination with scaffolds in tissue engineering in vivo studies. A hydrogel scaffold consisting of collagen type I and PRP was demonstrated to improve cellular recruitment of dermal-derived stem cells from fresh skin tissue and to accelerate wound healing in mice [181]. Another study showed that micro *β*-tricalcium phosphate (*β*-TCP) mixed with autologous platelet-derived growth factor material was able to create a lasting three-dimensional soft tissue augmentation in the cheek of mice 8 weeks after implantation [182]. Histological analysis revealed that many actively secreting fibroblasts were observed in the periphery of the *β*-TCP and that there were well differentiated fibroblast cell lines and blood vessels, with no signs of inflammation [183].

The use of PRP in tissue engineering studies aimed at increasing proliferation and differentiation of ASCs in combination with scaffolds. For osteoconductive purposes, ASCs were favoured to differentiate into osteogenic and endothelial cells when seeded in PRP-loaded gelatin-nanohydroxyapatite fibrous scaffolds, and the scaffold containing PRP gel performed better than those containing PRP powder [184]. Scioli et al. [185] documented that combined treatment with PRP and insulin favours the chondrogenic/osteogenic differentiation of ASCs in three-dimensional (3D) collagen type I scaffolds. For wound-healing purposes, Bayati et al. [186] verified that ASCs seeded on electrospun polycaprolactone fibers showed an increased proliferation rate and expression level of cytokeratin 14, filaggrin, and involucrin as compared to culture dishes and reconstituted a thick epidermal layer with several skin appendages in a wound-healing model in vivo. Our group showed that emulsification of subcutaneous fat and collagenase digestion allowed to obtain an adequate number of SVF cells that were seeded onto Integra® (1 × 10^6^ nucleated cells onto 1 × 1 cm pieces) in the presence of autologous PRP. After 7 days, cells started to form lacunar structures that were both CD31 and *α*-SMA positive (unpublished results), indicating that SVF cells were induced by growth factors produced by themselves and by PRP to generate the structure of new vessels. Other studies are needed to better understand which signals, coming either from scaffold glycosaminoglycans of scaffold or PRP, are involved in this kind of differentiation. The potential capability of ADSCs to differentiate in vessel-like structure will improve the regenerative capacity of dermal substitute, decreasing healing time in patient with chronic lesion of the lower limbs.

## 5. Conclusions

Nonhealing skin wounds are commonly related to peripheral vascular disease, infection, trauma, neurologic, and immunologic conditions, as well as neoplastic and metabolic disorders, and do represent a challenging problem in the medical practice. The injection of free fat associated with SVF/ASCs represents an alternative strategy to increase the healing rate of hard-to-heal wounds. The improvement seen by the usage of this technique has been mainly attributed to the enhancement of angiogenesis, while the secretion of growth factors improves tissue survival. These effects have been implemented in the tissue engineering field, with recent studies highlighting the differentiation capacity of SVF/ASCs within dermal matrix and collagen scaffolds for soft and hard tissue defect healing. The optimal scaffold to deliver SVF/ASCs has not been identified yet, and it may be dependent on different applications. Currently, the use of autologous additives such as PRP seems a promising approach in enhancing the applications of ASCs. PRP is nowadays considered a factory of growth factors and has been found to be useful for the treatment of patients affected by lower extremity ulcers and chronic wounds. Early clinical studies have confirmed the outcomes of preclinical experiences, setting the basis for further confirmation by means of larger, randomized, controlled, double-blind trials. Long-term preclinical and clinical studies in humans are also warranted, to exclude the possibility of emergence of tumoral growth.

## Figures and Tables

**Table 1:** Phenotypic markers of undifferentiated ASCs.

Marker	Expression	Reference
*Adhesion molecules*		
*β*1 integrin (CD29)	+	[26, 39]
*α*4 integrin (CD49d)	+	[13, 26]
Vascular cell adhesion molecule (VCAM; CD106)	+/-	[13, 26]
Intercellular adhesion molecule (ICAM-1; CD54)	+	[26]
Activated leukocyte adhesion molecule (ALCAM; CD166)	+	[26]
Tetraspan protein (CD9)	+	[26]
Endoglin (CD105)	+	[26]
CD34	+	[13, 26, 29]
*Receptors*		
Hyaluronan receptor (CD44)	+	[13, 26]
Transferrin receptor (CD71)	+	[13]
Triiodothyronine (T3) receptor *α*	+	[40]
*Surface enzymes*		
Neutral endopeptidase (CD10)	+	[26]
Aminopeptidase (CD13)	+	[26]
Ecto-5′-nucleotidase (CD73)	+	[40]
*Cytoskeleton proteins*		
*α*-Smooth muscle actin	+	[13, 26]
Vimentin	+	[24]
*Others*		
HLA-ABC	+	[26]
Decay accelerating factor (CD55)	+/-	[13, 26]
Protectin (CD59)	+	[13, 26]

+: positive association; -: negative association.

**Table 2:** Main animal models and results in wound healing.

Animal model	Wound healing	Biological effects	References
*Diabetic wounds*			
Full-thickness dermal wound in diabetic (*db/db*) mice	Multicellular aggregates of human ASCs > ASCs in suspension: rate of wound healing	Multicellular aggregates of human ASC > ASC in suspension: production of extracellular matrix proteins (tenascin C, collagen VI *α*3, and fibronectin) and secretion of soluble factors (HGF, MMP-2, and MMP-14)	[100]
Full-thickness circular excisional wound in nondiabetic and diabetic rats (streptozotocin-induced)	ASCs > untreated controls: rate of wound healing	No significant difference between volume density of collagen and vessel and also length density of vessels in ASC-treated and control groups	[101]
Excisional wound healing in normal and diabetic rats (streptozotocin-induced)	Rat ASCs significantly accelerated wound closure in normal and diabetic rat, including increased epithelialization and granulation tissue deposition	Increased VEGF, HGF, and FGF-2 protein expression in ASC-treated wounds, as compared with control and fibroblast-treated wounds	[102]
Full-thickness excision wound in diabetic (NOD/SCID; streptozotocin-induced) mice	Human ASC < human AM-MSCs: promotion of wound healing, reepithelialization, and cellularity	Human ASC < human amnion-derived MSCs: mRNA and protein expression of angiogenic factors (IGF-1, EGF, and IL-8)	[104]
Full-thickness excision wound in mice	Human ASC > BM-MSCs > AM-MSCs > untreated control: promotion of wound healing, reepithelialization, and granulation tissue	Human ASC > AM-MSCs and BM-MSCs > control: promotion of human DF migration. hDFs cocultured with ASC significantly upregulated the mRNA expression of VEGF, bFGF, KGF, and TGF-*β*	[105]
Full-thickness skin graft model in diabetic rats (induced by streptozotocin)	Autologous rat ASCs increased survival, angiogenesis, and epithelialization; reduced necrosis, as compared with untreated controls	ASCs increased VEGF and TGF-*β*3 expression in epidermis-dermis and graft-bed fascial area as compared with controls	[106]
Full-thickness wound made by biopsy punch in normal and diabetic (*dbdb*) mice	Mouse normal ASCs > mouse diabetic ASCs: wound healing rate; reepithelization and keratinocyte proliferation; granulation tissue formation; dermal regeneration	ND	[107]
Full-thickness wound in diabetic rats (streptozotocin-induced)	Rat ASCs accelerated wound healing as compared with diabetic rats without ASC treatment; reduced periwound inflammation; promoted cell proliferation	Rat ASCs increased the expression of EGF and VEGF in fibroblasts and endothelial cells at the wound margin	[115]
*Radiation wounds*			
Radiation-induced skin ulcer in rats	Rat ASCs promoted a faster rate of wound healing and increased neovascularization and granulation tissue as compared with untreated controls	ND	[112]
*Burn wounds*			
Partial-thickness scald injury in mice	Mouse ASCs alone or combined fat isografts and ASCs determined significantly decreased wound depth compared to fat isografts and untreated controls	ASCs alone or fat isograft with ASCs determined a significant reduction in apoptosis and increased vascularization by immunohistochemistry when compared to fat alone and controls	[110]
Full-thickness burn wounds in athymic mice created by thermal injury	No effect on wound healing time between ASC-treated and untreated cases; increased vascularity in ASC-treated mice	Increased type I collagen and type III collagen, and markers of adipogenesis (FABP-4, PPAR*γ*) in the ASC-treated group by RT-PCR analysis	[111]
*Surgical wounds*			
Full-thickness wound in rabbits	Autologous ASC inoculation induced a more rapid and more complete wound-healing process when compared with autologous BM-MSCs and allogeneic ASCs	ASC-treated wounds exhibited better regeneration of epithelial layers, collagen deposition, and PCNA-positive nuclei in epithelial regenerated epidermis compared to BM-MSC treated lesions	[113]
Full-thickness wound in mice	Clusters (speroids) of hASCs with low-level light therapy (LLLT) groups accelerated wound closure, including neovascularization and regeneration of skin appendages, compared with the other groups (cluster or LLLT)	hASC cluster was CD31^+^, CD34^+^, and KDR^+^. At the level of wound bed, greater amount of growth factors were observed in the cluster+LLLT group than in the control groups	[116, 117]

AM-MSCs: amniotic membrane-derived mesenchymal stem cells; ASCs: adipose-derived stem cells; bFGF: basic fibroblast growth factor; BM-MSCs: bone marrow-derived mesenchymal stem cells; EGF: epidermal growth factor; FABP: Fatty Acid Binding Protein; HGF: hepatocyte growth factor; IGF: insulin-like growth factor; IL: interleukin; KGF: keratinocyte growth factor; MMP: matrix metalloproteinase; PCNA: Proliferating Cell Nuclear Antigen; PPAR: peroxisome proliferator-activated receptor; TGF: transforming growth factor; VEGF: vascular endothelial growth factor.

**Table 3:** Use of ASCs and dermal substitutes in in vivo wound models.

Characteristics of ASCs	Dermal substitute	Study model	Model	Reference
ASCs	Acellular dermal matrix (ADM)	Skin injury model in mice	ASCs survived after in vivo engraftment, spontaneously differentiated along vascular endothelial, fibroblastic and epidermal epithelial lineages, and significantly improved wound healing	[151]
ASCs	Silk fibroin-chitosan (SFCS) scaffold	Full-thickness skin defect in male athymic mice	The extent of wound closure and microvessel density were significantly enhanced in the ASC-SFCS group versus SFCS	[152]
Freshly isolated murine ASCs	Atelocollagen matrix (ACM)	Full-thickness skin defect in diabetic mice	Advanced granulation tissue formation, capillary formation, and epithelialization in diabetic healing-impaired wounds treated with autologous ASC-containing ACMS, compared with mice treated with ACMS alone	[153]
ASCs	Acellular dermal matrix (ADM)	Subcutaneous implants for soft tissue augmentation	The thickness of the implanted material and the vascular density were the highest 8 weeks postoperatively in ASC-seeded ADM as compared with ADM without ASCs	[154]
Cultured ASCs	Small intestinal submucosa (SIS); acellular dermal matrix (ADM); composite scaffold (collagen-chondroitin sulfate-hyaluronic acid (Co-CS-HA))	Murine skin injury model	ASC-seeded scaffolds enhanced the angiogenesis and wound-healing rate compared with the nonseeded scaffolds; SIS and ADM promoted higher vascularity than Co-CS-HA scaffolds	[155]
Freshly isolated ASCs	Integra®	Rat model of skin wound	Increased vascularization and collagen deposition after 1-3 weeks the implant with ASCs was seeded	[156]
Freshly porcine ASCs	Integra®	Full thickness thermal burns in swine	Accelerated maturation of wound bed tissue, significant increase in depth of the wound bed tissue, collagen deposition, and blood vessel density in wounds receiving ASC-loaded scaffolds compared to vehicle-loaded scaffolds	[157]
Cultured murine ASCs	Acellular dermal matrix (ADM)	Excisional wound-healing model in diabetic rats	Capillary density was evidently increased in the ASC–ADM group compared with the control or the ADM group, resulting in accelerated wound closure	[158]
Freshly isolated ASCs	Bilayer and Flowable Integra® scaffolds	Grafting of scaffolds in the dorsum of nude mice	Increased neovascularization and formation of new connective tissue (loose and adipose)	[159]
Cultured ASCs	Decellularized dermal matrix prepared from mouse skin	Full-thickness cutaneous wound in nude mice	Increased granulation thickness, reepithelization, blood vessel density	[160]
Freshly isolated ASCs	Bioengineered pigmented dermoepidermal skin substitutes (melDESS), composed of dermal fibroblasts, keratinocytes, melanocytes, and ASCs	melDESS transplanted on the backs of immunodeficient rats	Decreased melanin synthesis and, consequently, greatly reduced pigmentation of melDESS	[161]
Cultured ASCs	Silk fibroin (SF)/chitosan (CS) film	Wound in diabetic rats	Wound healing was drastically enhanced for ADSC-SF/CS treatment groups compared with control groups and SF/CS film treatment groups	[162]
ASCs grown in 10% human plasma	Human acellular dermal matrix (Gliaderm®)	Full-thickness dorsal wounds in immunodeficient mice	Granulation thickness, vascularization, and reepithelialization were significantly increased, resulting in complete wound healing in 12 days	[163]
